# Influence of E-Liquid Humectants, Nicotine, and Flavorings on Aerosol Particle Size Distribution and Implications for Modeling Respiratory Deposition

**DOI:** 10.3389/fpubh.2022.782068

**Published:** 2022-03-17

**Authors:** Aleksandr B. Stefaniak, Anand C. Ranpara, Mohammed Abbas Virji, Ryan F. LeBouf

**Affiliations:** Respiratory Health Division, National Institute for Occupational Safety and Health, Morgantown, WV, United States

**Keywords:** electronic cigarette, vaping, secondhand or passive exposure, occupational exposure, cascade impactor

## Abstract

Electronic cigarette, or vaping, products are used to heat an e-liquid to form an aerosol (liquid droplets suspended in gas) that the user inhales; a portion of this aerosol deposits in their respiratory tract and the remainder is exhaled, thereby potentially creating opportunity for secondhand exposure to bystanders (e.g., in homes, automobiles, and workplaces). Particle size, a critical factor in respiratory deposition (and therefore potential for secondhand exposure), could be influenced by e-liquid composition. Hence, the purposes of this study were to (1) test the influence of laboratory-prepared e-liquid composition [ratio of propylene glycol (PG) to vegetable glycerin (VG) humectants, nicotine, and flavorings] on particle size distribution and (2) model respiratory dosimetry. All e-liquids were aerosolized using a second-generation reference e-cigarette. We measured particle size distribution based on mass using a low-flow cascade impactor (LFCI) and size distribution based on number using real-time mobility sizers. Mass median aerodynamic diameters (MMADs) of aerosol from e-liquids that contained only humectants were significantly larger compared with e-liquids that contained flavorings or nicotine (*p* = 0.005). Humectant ratio significantly influenced MMADs; all aerosols from e-liquids prepared with 70:30 PG:VG were significantly larger compared with e-liquids prepared with 30:70 PG:VG (*p* = 0.017). In contrast to the LFCI approach, the high dilution and sampling flow rate of a fast mobility particle sizer strongly influenced particle size measurements (i.e., all calculated MMAD values were < 75 nm). Dosimetry modeling using LFCI data indicated that a portion of inhaled particles will deposit throughout the respiratory tract, though statistical differences in aerosol MMADs among e-liquid formulations did not translate into large differences in deposition estimates. A portion of inhaled aerosol will be exhaled and could be a source for secondhand exposure. Use of laboratory-prepared e-liquids and a reference e-cigarette to standardize aerosol generation and a LFCI to measure particle size distribution without dilution represents an improved method to characterize physical properties of volatile aerosol particles and permitted determination of MMAD values more representative of e-cigarette aerosol *in situ*, which in turn, can help to improve dose modeling for users and bystanders.

## Introduction

Electronic cigarette, or vaping, products (e-cigarettes) heat a liquid (e-liquid) to form an aerosol that the user inhales. Since their introduction, the internal design and external appearance of e-cigarettes has evolved continuously (1–3). Currently, there are four “generations” of e-cigarettes that differ in external appearance and internal design (4, 5); however, all generations have in common: (1) a battery, (2) a chamber that contains a heating coil, (3) a cartridge to store the e-liquid, and (4) a mouthpiece through which the user inhales (6). When the user inhales through the device, the battery heats the coil that is wrapped in a wick, which is wetted with e-liquid from the cartridge, and vaporizes the e-liquid; as it travels to the mouthpiece, the vapor cools and condenses to form an aerosol (liquid droplets suspended in gas) that is delivered to the respiratory tract (7, 8).

E-liquids are composed of humectants, and sometimes water, ethanol, flavorings, and/or nicotine (9). Humectants are hygroscopic substances that retain moisture and constitute the main ingredients in e-liquids (10, 11). Propylene glycol (PG) and/or vegetable glycerin (VG) are the most common humectants, and their relative proportions in an e-liquid can be tailored to the user's personal experiences and preferences (2, 7, 10). Water and ethanol are added to the humectants as diluents for flavorings (11). Flavorings are added to the e-liquid to impart taste and aromas to the inhaled aerosol (11–14). Nicotine, when present, is in either the free-base (basic pH ~8–10) or salt (acidic pH) form; e-liquids used in third and prior generation e-cigarettes contained up to 95% of their total nicotine in free-base form (15), whereas e-liquids for fourth generation e-cigarettes contain nicotine in the acidic salt form (16–18).

Upon inhalation of aerosol generated by an e-cigarette, a portion of the particles (liquid droplets) and gases deposit throughout the respiratory tract of the user and the remainder is exhaled (19, 20). This exhaled portion creates potential for secondhand exposures among persons in proximity to e-cigarette users. Secondhand exposures can occur in home environments (5, 21–23) and in occupational environments that span a range of industries such as hospitality venues (e.g., convention centers), bars, restaurants, and nightclubs as well as businesses adjacent to e-cigarette retail stores that permit their use indoors (24–29). E-cigarette aerosol that settles onto surfaces in homes, vehicles, or workplaces can serve as a source of dermal exposure from skin contact with residues (30, 31).

Particle size, in part, influences where e-cigarette aerosol liquid droplets will deposit in the respiratory tract (8, 19, 32–35). Hence, understanding factors that influence particle size distribution (PSD) of aerosol generated by e-cigarettes are needed to model dosimetry. These factors can be broadly categorized as consumable-related (e.g., e-liquid chemical composition), device-related (e.g., e-cigarette power setting), and user-related (e.g., puff topography). The focus of the current research is on the influence of consumable-related factors on aerosol PSD, specifically humectants, nicotine, and flavorings. Literature on the influence of these e-liquid constituents on PSD are conflicting. Some studies reported that particle size decreased as the percentage of PG in the e-liquid increased (4, 7, 33, 36–38), whereas other studies indicated that PSD increased as the percentage of PG in the e-liquid increased (39), or was not influenced by the ratio of PG and VG in the e-liquid (35, 40). The presence of nicotine in e-liquids is reported to increase particle size (33, 41, 42), decrease particle size (41), or have no effect (8, 43–47). Several studies reported that aerosol characteristics were not influenced by the presence of flavorings in e-liquids (8, 33, 35, 45, 47, 48), though one study reported that the presence of vanillin drastically increased particle size. Note that this inter-comparison of reviewed literature results is difficult because observed inconsistencies could reflect that the commercially available e-liquids contained undisclosed ingredients or ingredients with unknown purity that influenced PSD and/or that other critical factors that influenced PSD were not consistent among prior studies.

The primary purpose of this study was to evaluate the hypothesis that e-liquid composition (humectant ratio, flavorings, and nicotine) influenced aerosol particle size. To test this hypothesis, laboratory-prepared e-liquids and a reference second-generation e-cigarette were used to generate aerosols. A cascade impactor was used to measure the mass-based aerosol PSD and real-time instruments were used to monitor number-based PSD during puffing. The secondary purpose of this work was to use the PSD data to model the regional deposition of aerosol particles in the respiratory tract of e-cigarette users. An estimate of the exhaled fraction of aerosol was derived from these modeling results as an indicator of potential for secondhand exposure.

## Materials and Methods

Existing literature indicated that the PG:VG humectant ratio of e-liquids can range from 100:0 to 0:100 (9, 49). PG:VG ratios of 70:30 and 30:70 are commonly used to evaluate size distribution (33) and toxicity (50) of e-cigarette aerosols. An analysis of 800 commercially available e-liquids indicated that the total volume fraction of water and ethanol never exceeded 10% in the products, i.e., either 10% water, 10% ethanol, or 5% water and 5% ethanol (11). E-liquids for third and prior generation e-cigarettes contained ~1–4% (10–40 mg/mL) flavorings and 0.6–3% nicotine (6–30 mg/mL), though current fourth generation e-cigarettes can contain 5–7% (50–70 mg/mL) nicotine (51, 52). This wide variability in composition (and ambiguity of ingredient purity and presence of undisclosed ingredients) of commercially available e-liquids can present challenges for experimental studies to elucidate the influence of specific constituents on aerosol particle size. As such, for experimental investigations, “model” or “reference” e-liquids with proportions that mimic commercially available products that are prepared in the laboratory under controlled conditions are useful surrogates (recognizing that an ideal laboratory reference e-liquid has less “real-world” generalizability than commercially available products) (11, 53, 54). Hence, for the current study, we chose to use laboratory-prepared e-liquids composed of varying ratios of humectants with water and ethanol and/or flavorings and nicotine in accordance with the Association Française de Normalization (AFNOR) standard D90-300-2 (55). The sources and purity of reagents were as follows: PG (ACS grade, CAS# 57-55-6, Fisher Scientific, Pittsburgh, PA, USA), VG (Certified ACS grade, CAS# 56-81-5, Fisher Scientific), 200 proof ethanol (ACS/USP grade, CAS# 64-17-5, Pharmaco-Aaper, Brookfield, CT), nicotine (>99% GC grade liquid, CAS# 54-11-5), vanillin (99%, CAS# 121-33-5), 3-methyl-1-butanol (99%, CAS# 123-51-3), 2-methylbutyric acid (98%, CAS# 1730-91-2), 2,3-butanedione (99%, CAS# 431-03-8), 2,3-pentanedione (97%, CAS# 600-14-6), and 2,3-hexanedione (99%, CAS# 3848-24-6) flavorings (all from ACROS Organics™, Geel, Belgium). Two proportions of PG and VG humectants (30:70, 70:30) with 18 milli-Ohm (MΩ) water (1% w/w) and 200 proof ethanol (1% w/w) but no nicotine or flavorings were prepared gravimetrically using a microbalance with mass resolution of 0.1 mg (XS 250, Mettler-Toledo LLC, Columbus, OH, USA). Each e-liquid was vortexed for 1 min to mix. To evaluate the influence of nicotine on PSD, the humectants e-liquids were also prepared with nicotine (2.4% w/w). To evaluate the influence of flavorings on PSD, 1% (w/w) of each flavoring (vanillin, 3-methyl-1-butanol, 2-methylbutyric acid, 2,3-butanedione, 2,3-pentanedione, and 2,3-hexanedione) was dissolved in PG then diluted with VG to achieve PG:VG ratios of 30:70 and 70:30; the final concentration of each flavoring in the e-liquids prepared in this manner was 0.3% w/w. E-liquids prepared with nicotine or flavorings were homogenized for 1 h using a rotator (Model 4152110, Thermo Scientific, Dubuque, IA) to mix.

### Aerosol Generation

To achieve comparable and repeatable aerosol generation, an automated E-cigarette Aerosol Generator (ECAG; e ~ Aerosols, LLC, Central Valley, NY, USA) was used to control power delivered to the e-cigarette heating coil and maintain a consistent coil temperature. The ECAG works on positive pressure to aerosolize an e-liquid at a user-defined puff topography by heating the coil at 3.7 V (set) and 1.6 A (measured) of electric current. Six measurement trials were performed for each e-liquid. For each trial, 1.2 mL of e-liquid was added to the chamber (tank) of an NJOY top tanks (NJOY, Inc., Scottsdale, AZ) second-generation reference e-cigarette from the NIDA Drug Supply Program (56). A separate new NJOY chamber was used for each e-liquid formulation (i.e., one new NJOY chamber was used for all six trials with 30:70 PG:VG, a separate new chamber was used for all trials with 70:30 PG:VG, and so on). The puff topography was set to 55 ml puff volume for 3 s (1 puff) with a 30 s puff delay. For each e-liquid, 2 puffs were generated per trial to measure the mass-based PSD using a low flow cascade impactor (LFCI) and ~30 puffs were generated per trial to measure the number-based PSD using mobility analyzers (57). The mass of e-liquid in the e-cigarette chamber was weighed on the microbalance prior to and after each trial.

### Aerosol Characterization

Accurate measurement of e-cigarette aerosol characteristics (mass, number, size) is challenging because (1) a high droplet number concentration is generated during each puff, (2) some droplets contain constituents that are highly volatile, and (3) humectants are hygroscopic (8, 19, 36, 43, 58–60). Given the presence of volatile constituents, e-cigarette aerosol properties can change because of evaporation within aerosol sampling instruments, which in turn, can significantly distort PSD measurements (61). Hence, for e-cigarettes, the choice of measurement approach is an important consideration in the experimental design. To date, various approaches have been utilized and included real-time instruments and cascade impactors (4, 7, 8, 36, 58, 62, 63). Real-time instruments such as mobility sizers operate at high sampling flow rates and often require dilution of the aerosol to bring the number concentration within the measurement range of the instrument; both high sampling flow rate and high dilution can promote evaporation of aerosol droplets, thereby introducing bias into PSD measurements (7, 8, 35, 36, 63). In contrast, cascade impactors are generally not affected by high particle number concentrations, and if a low-flow impactor is used for aerosol collection and its sampling flow rate is closely matched to the e-cigarette puff flow rate, evaporative losses from dilution can be minimized, and PSD determined more accurately. Additionally, since the impactor stages must be analyzed gravimetrically, further losses of very volatile constituents can be minimized by quickly measuring mass or by applying an experimentally derived correction factor (4, 7, 8, 35, 36, 58, 63). For additional details on the relative advantages and disadvantages of sampling e-cigarette aerosols, the reader is referred to prior literature (8, 35, 64). For the purposes of this study, a LFCI was used to characterize aerosol mass-based size distribution to understand the influence of e-liquid constituents on PSD and to model aerosol deposition in the respiratory tract. Real-time mobility sizers were used to measure aerosol number concentration and the data used to calculate PSD values for comparison to the impactor results.

### Determination of Mass-Based Aerosol Particle Size Distribution

**Figure 1:**
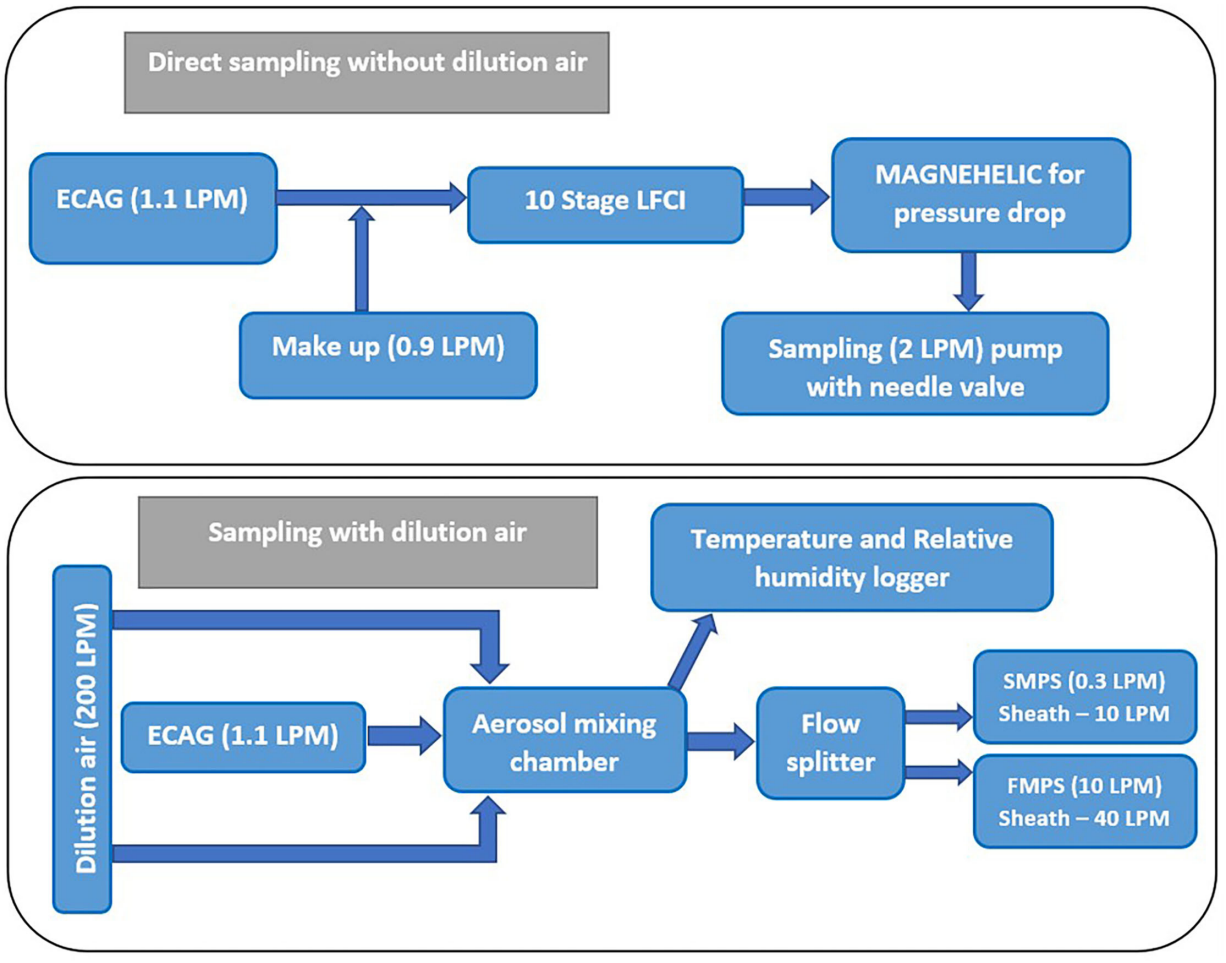
Schematic of test system for measurement of e-liquid aerosol mass- **(top)** and number- **(bottom)** based particle size distributions. ECAG, e-cigarette aerosol generator; FMPS, fast mobility particle sizer; LFCI, low-flow cascade impactor; LPM, liters per minute; SMPS, scanning mobility particle sizer.

A LFCI (MiniMOUDI™, TSI Incorporated, Shoreview, MN, USA) with 37-mm diameter aluminum collection substrates (Fisher Scientific) was used to size-separate the aerosol generated by the e-cigarette into 10 size fractions (d_50_ cut-points = 0.056, 0.1, 0.18, 0.32, 0.56, 1.0, 1.8, 3.2, 5.6, and 10 μm) at the default sampling flow rate of 2 liters/min (LPM). Prior to sampling, the NJOY e-cigarette was filled with 1.2 mL of e-liquid and puffed for 10 min to condition the e-cigarette and ECAG system. Aerosol from each e-liquid was sampled directly into the LFCI without further dilution by connecting the inlet of the impactor to the e-cigarette mouthpiece using flexible, black conductive silicone tubing that was 70 cm long with 0.5 cm (inner diameter) to minimize aerosol wall losses. To maintain a constant sampling flow rate of 2 LPM for the cascade impactor, a high efficiency particulate air (HEPA)-filtered air bypass was used to provide 0.9 LPM of laboratory air during puffing with 1.1 LPM air per puff provided by the ECAG. During the inter-puff interval, the same calibrated sampling pump provided 2 LPM air flow from the HEPA-filtered bypass to the impactor (Figure 1). After the last puff of a trial, the mass of aerosol collected on each pre-weighed aluminum substrate was quickly determined using a microbalance (XS 250, Mettler-Toledo) within minutes of sampling to minimize evaporation. For each trial, the mass median aerodynamic diameter (MMAD) and geometric standard deviation (GSD) were calculated from the log-transformed gravimetric measurements of sample mass collected on each stage of the LFCI using a probit model as described previously (65).

To verify that the LFCI impactor flow rate and gravimetric weighing procedure did not substantially bias PSD determinations, a mass loss experiment was performed (*n* = 3 trials) in which three puffs per e-liquid were collected with the impactor and masses were measured 1, 5, and 15-min post-collection all while 2 LPM of air flowed through the impactor. For purposes of this experiment, only the masses of e-liquid aerosol collected on the impactor stages with d_50_ cut-points of 0.32, 0.56, and 1 μm were measured as these stages accounted for more than 89% of the mass deposited in the impactor for all e-liquids and trials. This observation that most mass was limited to a few impactor stages was consistent with Baassiri et al. who reported that 76% of the aerosol mass collected in their study was on LFCI stages with d_50_ cutoffs of 0.5–2.5 μm (7). For each e-liquid, the total mass loss among the three impactor stages combined did not exceed 10% (Supplementary Figure 1). Therefore, no correction factor was applied to the MMAD estimates for each e-liquid formulation.

### Determination of Number-Based Aerosol Particle Size Distribution

A scanning mobility particle sizer spectrometer (SMPS; Model 3080, TSI Inc., Shoreview, MN, USA) and fast mobility particle sizer spectrometer (FMPS; Model 3091, TSI Inc.) were used to continuously measure mobility size during e-liquid aerosolization. The SMPS is capable of measuring particles in the size range 2–1,000 nm in 128 channels with scan time of 195 s at a sampling flow rate of 0.3 LPM and sheath flow rate of 10 LPM. The FMPS is capable of measuring particles with size 5.6–560 nm in 32 channels on a second-by-second basis at a sampling flow rate of 10 LPM and sheath flow rate of 40 LPM. To permit inter-comparison of data, the measurement size range of the SMPS was truncated to 6–560 nm to match the FMPS and the integrating time for the FMPS was adjusted to 195 s to match the SMPS. The aerosol count median diameter (CMD) for each trial was obtained from the instrument software. Prior to measuring e-cigarette aerosol, the inlet of each instrument was connected to a high-efficiency particulate air filter (HEPA-CAP™ 36, GE Healthcare Whatman™, Fisher Scientific) to verify operability. To bring the aerosol number concentration within the measurement range of these instruments, the aerosol generated by the ECAG was diluted with ~200 LPM of HEPA-filtered air. Using a flow splitter, the SMPS and FMPS were connected to the diluter and sampled air for at least 5 min to measure background particle concentrations, next the ECAG was turned on and the e-cigarette with an e-liquid was puffed (same topography as for mass measurements) for 1 h, followed by post-puffing background air monitoring for 5 min. Six trials were conducted for each e-liquid per mobility sizer instrument. The mass median diameter (MMD) was calculated from the average CMD value for each e-liquid trial using the Hatch-Choate equation (Equation 1), moment average, b = 3, and geometric standard deviation, σ_g_:


(1) $$\begin{array}{r} \text{MMD}={\text{CMD}}^{*}\exp\left({\text{b}}^{*}\ln^{2}\sigma_{g}\right). \end{array}$$

Values of the MMAD were calculated by multiplying the MMD by the square root of the weighted average density of humectants in the e-liquids (70:30 PG:VG = 1.11 g/cm^3^, 30:70 PG:VG = 1.19 g/cm^3^).

Using Equation 2 below, the mass of aerosol collected per LFCI stage (*M*_*stage*_), in units of μg/cm^3^ was calculated as the difference in mass of the aluminum substrate after sampling (*M*_*post*_) compared with its mass before sampling (*M*_*pre*_) divided by average ECAG sampling volume (*V*^*ECAG*^). The total aerosol mass concentration for each e-liquid formulation was calculated by summing the calculated concentration values for all impactor stages for a trial.


(2) $$\begin{array}{r} M_{stage}={\frac{\;M_{post}\left(mg\right)-M_{pre}\;\left(mg\right)}{{\overset{\bar{}}{V}}_{ECAG}\;\left(cm^{3}\right)}}^{*}\frac{1000\;\mu g}{mg} \end{array}$$

### Dose Modeling

Knowledge of particle size, coupled with physiological data on respiratory tract characteristics (anatomy, ventilation parameters), can be used to model dosimetry for e-cigarette users. When known, the PSD of exhaled aerosol from e-cigarette users can be used to model dosimetry for bystanders. Several models exist for estimating particle deposition throughout the respiratory tract, including computational fluid dynamics (CFD) approaches, the International Commission for Radiological Protection (ICRP) human respiratory tract model, and the multiple path particle dosimetry model (MPPD) (34). Among these models, the MPPD model is based on realistic lung geometry, physiology and deposition mechanisms, and it provides estimates of both the whole-lung and the regional particle deposition fractions that were validated with experimental data (34). Hence, for our purposes, the freely available MPPD model (version 3.04, ARA) was used to conceptually estimate regional and total particle deposition throughout the respiratory tract of e-cigarette users and derive an estimate of the exhaled particle fraction.

The mass fraction of particles that could deposit in the head (H), tracheobronchial (TB), and pulmonary (P) regions were estimated using in MPPD using the Yeh/Schum symmetric lung model for an oronasal-mouth breather. This model was chosen rather than oral-only inhalation because available evidence indicates histological changes in the nasal cavity epithelial lining and oral mucosal damage among e-cigarette users (6, 66, 67). Supplementary Table 1 summarizes the details of the physiological parameters used for particle deposition modeling. The total amount of particles that could deposit in the respiratory tract was calculated by summing the H, TB, and P regional deposition fractions. The mass fraction of particles that could be exhaled by the e-cigarette user was conceptually estimated as 1—total deposited fraction, to provide a rough indicator of secondhand exposure potential (68).

It is important to note that the CFD, ICRP, and MPPD models are intended for reasonably diluted non-volatile particles; however, aerosol generated by e-cigarettes do not meet this condition because the high number concentration produced by a puff behaves as a “cloud” or bolus and droplets may change in size *via* coagulation during mouth hold and/or absorption of water, conductive heat and diffusive/convective vapor transport, and dilution/mixing as they travel throughout the respiratory tract, which in turn will affect estimates of the amount deposited in a given region (19, 20, 59). Hence, estimates of particle deposition in the respiratory tract and subsequent exhalation fraction provided herein are intended only to illustrate these concepts and the numerical values reported should be interpreted with caution.

### Statistical Analyses

Data acquired from LFCI trials (*n* = 6 per e-liquid type) were compared using least squares linear regression models and Tukey's HSD to account for multiple comparisons. Statistics were computed using JMP 13.0 and SAS 9.4 (SAS Institute Inc., Cary, NC) at α = 0.05 as the level of significance.

## Results

Table 1 summarizes the mass-based aerosol characteristics measured using a LFCI for each e-liquid. The mass of e-liquid consumed ranged from 2,578 μg/puff (30:70 PG:VG) to 3,971 μg/puff (70:30 PG:VG); amounts did not differ by e-liquid type. The average aerosol mass concentration per puff ranged from ~50 to over 90 μg/cm^3^. For the 70:30 PG:VG e-liquids, the presence of flavorings and nicotine resulted in significantly higher mass concentrations per puff compared with the 70:30 PG:VG e-liquid. In general, variability (coefficient of variation) in mass concentrations per puff were higher for 30:70 PG:VG e-liquids compared to the 70:30 PG:VG e-liquids. The presence of flavorings or nicotine influenced particle size, i.e., MMADs of aerosol from e-liquids that contained humectants only were significantly larger compared with aerosol from e-liquids that contained flavorings or nicotine (*p* = 0.005). MMADs were also influenced by the relative proportion of humectants. Specifically, aerosols from e-liquids prepared with 70:30 PG:VG had significantly larger MMADs compared with aerosol from e-liquids prepared with 30:70 PG:VG (*p* = 0.017).

**Table 1:** Influence of e-liquid formulation on mass-based aerosol characteristics measured using a low-flow cascade impactor (*n* = 6 trials/e-liquid).

	**E-liquid consumed (μg/puff)**		**Concentration per puff (μg/cm** ^**3**^ **)**			
**E-liquid**	**Mean ±SD**	**CV (%)**	**Mean ±SD**	**CV (%)**	**MMAD (μm)**	**GSD**
30:70 PG:VG	3,906 ± 1,233^A^	32	67.4 ± 21.6^A, B, C^	32	0.93^A, B^	1.43
30:70 PG:VG w/flavorings	2,578 ± 451.3^A^	18	51.0 ± 7.8^C^	15	0.88^B^	1.38
30:70 PG:VG w/nicotine	3,272 ± 220.6^A^	6.7	84.5 ± 22.7^A^	27	0.86^B^	1.36
70:30 PG:VG	3,931 ± 1,648^A^	42	51.7 ± 3.0^B, C^	6	1.00^A^	1.43
70:30 PG:VG w/flavorings	3,328 ± 284.7^A^	8.5	78.8 ± 5.3^A, B^	7	0.93^A, B^	1.36
70:30 PG:VG w/nicotine	3,597 ± 560.0^A^	16	90.3 ± 19.5^A^	22	0.91^A, B^	1.38

*SD, standard deviation; CV, coefficient of variation; MMAD, mass median aerodynamic diameter; GSD, geometric standard deviation; PG, propylene glycol; VG, vegetable glycerin*.

*Levels not connected by same letter (A, B, C) are significantly different. For main effects on comparisons of MMADs, the absence of flavoring or nicotine in the humectant significantly increased MMADs of aerosol from e-liquids (p = 0.005). Also, significantly increased MMADs were observed for 70:30 PG:VG compared with aerosol from e-liquids prepared with 30:70 PG:VG (p = 0.017)*.

**Figure 2:**
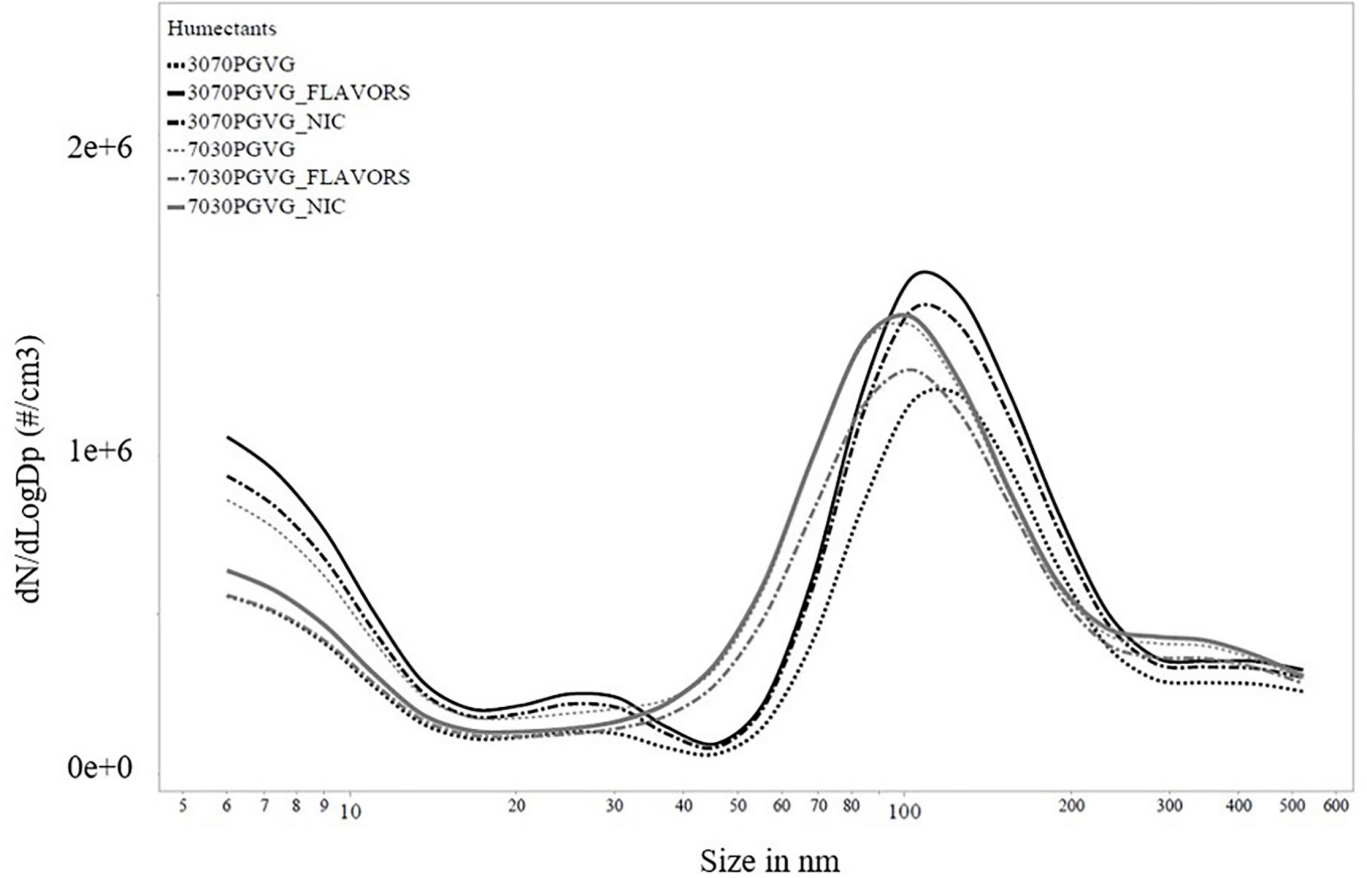
Particle number concentration by size for fast mobility particle sizer (FMPS) measurements. PG, propylene glycol; VG, vegetable glycerin; Nic, nicotine.

**Figure 3:**
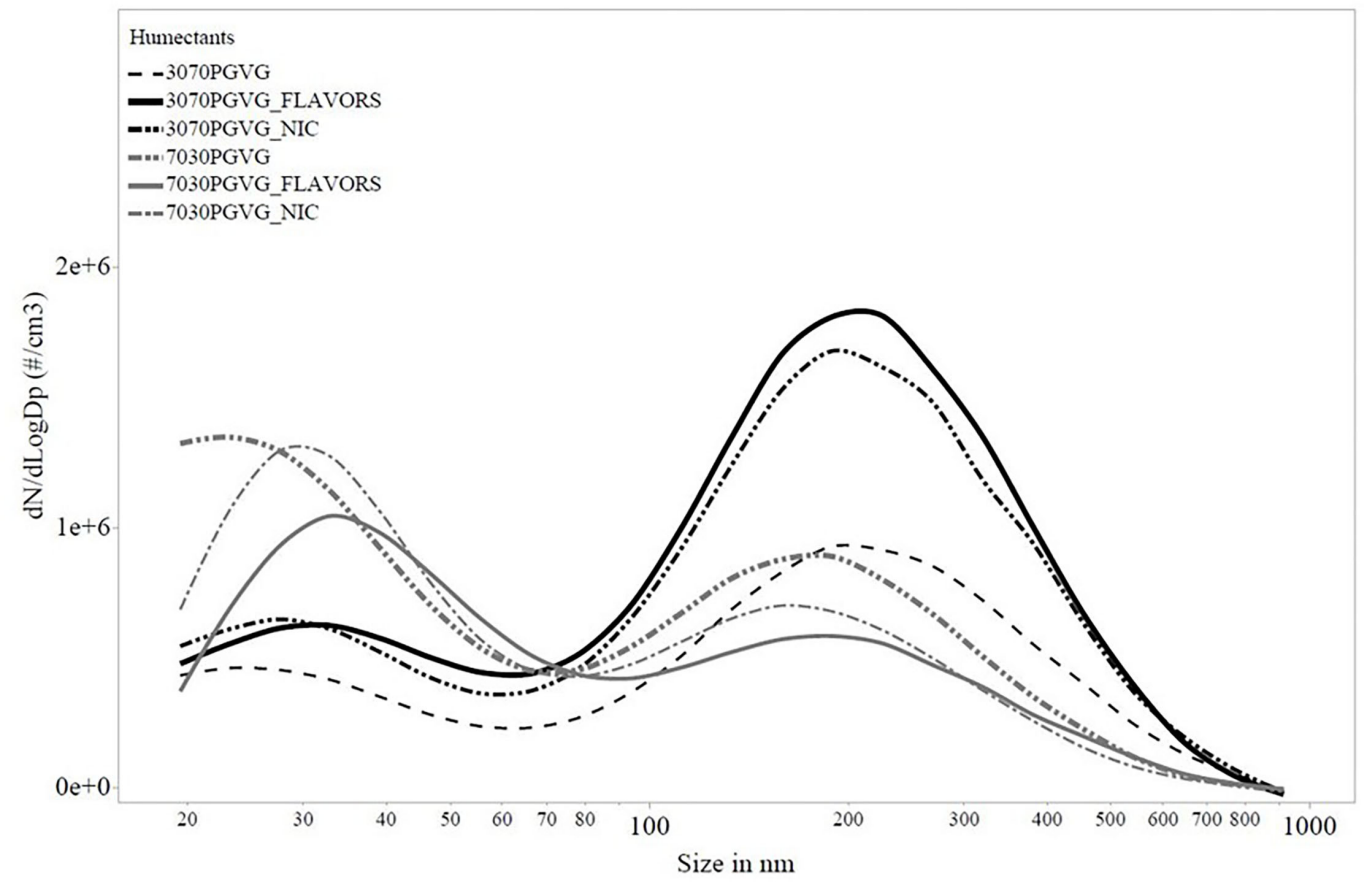
Particle number concentration by size for scanning mobility particle sizer (SMPS) measurements. PG, propylene glycol; VG, vegetable glycerin; Nic, nicotine.

Figures 2, 3 show the particle number concentration from FMPS and SMPS measurements, respectively. Major peaks were on the order of a few hundred nanometers for both instruments. As summarized in Table 2, CMD values from all mobility sizer measurements were below 0.15 μm. The MMAD values calculated from the FMPS data were all below 75 nm, and MMAD values calculated from the SMPS data ranged from 0.93 to 2.23 μm.

**Table 2:** Number and calculated mass-based aerosol characteristics from fast mobility particle sizer (FMPS) and scanning mobility particle sizer (SMPS) measurements (*n* = 6 trials/e-liquid).

	**FMPS**			**SMPS**		
**E-liquid**	**CMD (μm)**	**GSD**	**MMAD (μm)**	**CMD (μm)**	**GSD**	**MMAD (μm)**
30:70 PG:VG	0.075	1.004	0.082	0.132	2.6	2.23
30:70 PG:VG w/flavorings	0.060	1.005	0.066	0.141	2.4	1.54
30:70 PG:VG w/nicotine	0.062	1.005	0.068	0.140	2.4	1.52
70:30 PG:VG	0.064	1.004	0.067	0.076	2.6	1.24
70:30 PG:VG w/flavorings	0.073	1.004	0.077	0.078	2.5	1.02
70:30 PG:VG w/nicotine	0.072	1.004	0.076	0.071	2.5	0.93

*CMD, count median diameter; GSD, geometric standard deviation; MMAD, mass median aerodynamic diameter; PG, propylene glycol; VG, vegetable glycerin*.

**Figure 4:**
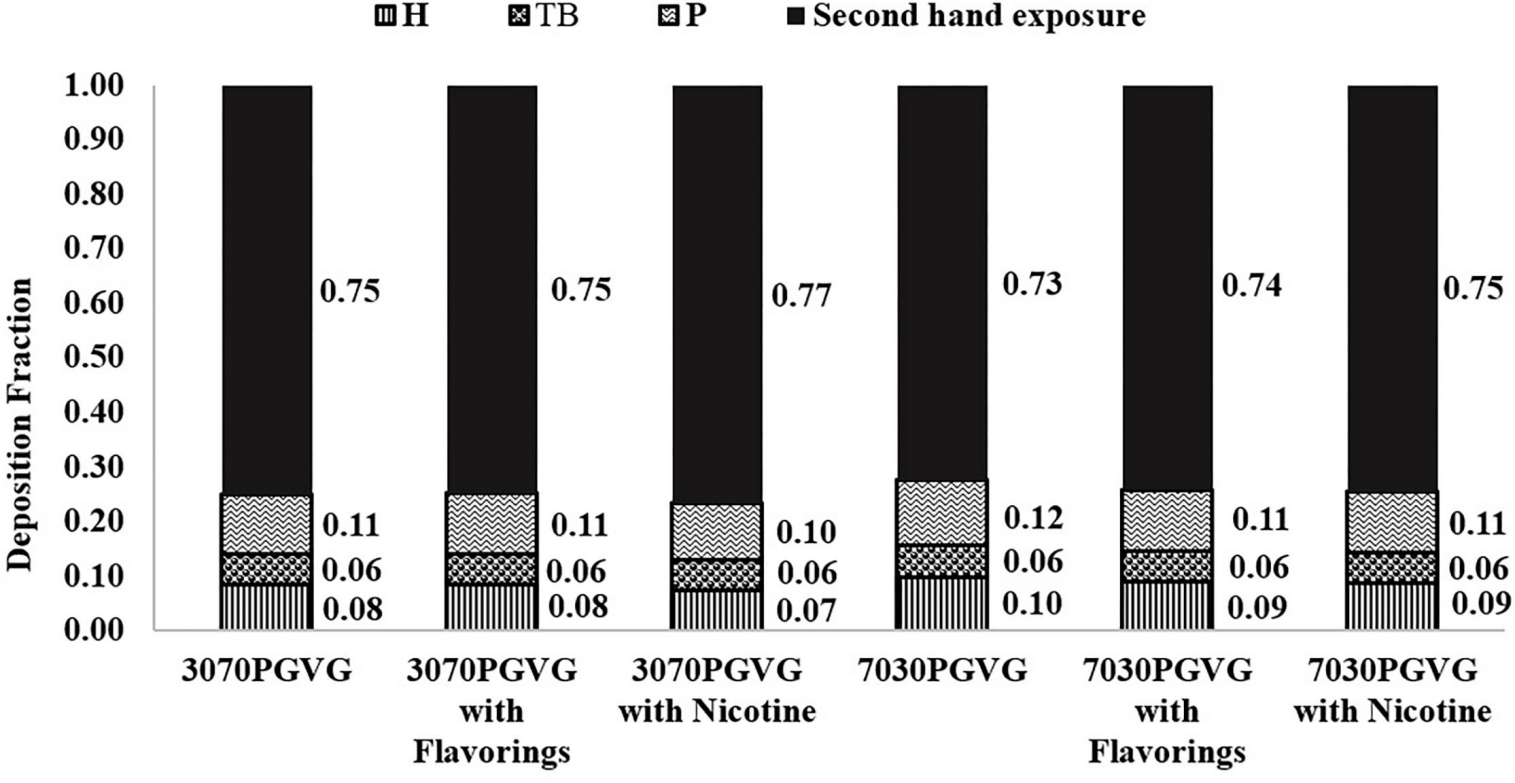
Regional and total aerosol deposition in e-cigarette users and exhaled fraction by e-liquid (low-flow cascade impactor data). H, head region; TB, tracheobronchial region; P, pulmonary region; PG, propylene glycol; VG, vegetable glycerin.

Figure 4 summarizes the regional and total particle deposition estimates in the respiratory tract for each e-liquid that was determined from the LFCI data and assuming a symmetrical lung model. Though significant differences were observed in MMAD values among some e-liquid formulations, regional and total deposition estimates were similar, i.e., ~8–10% of particles will deposit in the H region, 6% will deposit in the TB region, and 10–12% will deposit in the P region, with total deposition of 23–27%. Assuming that any undeposited aerosol is exhaled, the remainder of particles could contribute to potential secondhand exposure.

## Discussion

The primary purpose of this study was to evaluate the hypothesis that e-liquid composition (humectant ratio, flavorings, and nicotine) influenced aerosol particle size. To test this hypothesis, laboratory-prepared e-liquids and a reference second-generation e-cigarette were used to generate aerosols that were sampled using a LFCI to measure the mass-based aerosol PSD and real-time instruments to monitor number-based PSD during puffing. LFCI measurements indicated that humectant ratio influenced PSD of laboratory-prepared e-liquids aerosolized using a second-generation e-cigarette. In the current study, MMADs determined using a LFCI were larger for e-liquids that contained a higher proportion of PG (Table 1). This observed influence of PG on mass-based PSD is consistent with a prior report (39), but contrasts observations from other investigators that mass-based particle size measured using impactors decreased as the percentage of PG in the e-liquid increased (4, 7, 36, 58). The exact reason for this divergence in experimental results is unknown at this time but could be related to the hygroscopic and/or volatility properties of the humectant constituents (33). The presence of nicotine or flavorings yielded significantly smaller MMADs (LFCI data) compared with e-liquids composed of only humectants (Table 1). Larcombe et al. observed that for VG-based e-liquids, particle size measured *in situ* with an optical particle spectrometer was smaller in the presence of nicotine (41). Other reports indicated that particle size for e-liquids measured using an optical particle sizer or mobility particle sizers increased in the presence of nicotine (33, 41, 42). Some investigators have reported that the presence of nicotine had no effect on particle size measured using mobility and aerodynamic particle sizers or low-pressure impactors (8, 43–47). Fuoco et al. observed that flavorings did not influence PSD of aerosolized e-liquids (45), whereas Lechasseur et al. reported that the presence of vanillin dramatically increased number-based particle size (33). The reason for the observed inconsistent influence of vanillin on PSD between our study and Lechasseur et al. is unknown but could be related to the measurement methods and/or the concentration of vanillin in the tested e-liquids. In our study, the concentration of vanillin in e-liquids was 0.3%, whereas in the Lechasseur et al. study, the concentration of vanillin in e-liquids was 1%. It is difficult to interpret the meaning of similarities and differences in results from the current study with published literature. In the current study, e-liquids were prepared in the laboratory to maintain control over composition and a reference e-cigarette was used to facilitate future replication of our work. However, even with this standardization, inter-comparison of measurement among studies is complicated because of differences in e-cigarette device power settings, differences in experimental setups (e.g., amount of dilution air or puff topography), and differences in aerosol monitoring approaches (e.g., the same sampler or monitor operated under different conditions or use of different samplers and monitors that measure PSD based on some other principle).

MMADs measured using a LFCI ranged from 0.86 to 1.00 μm (Table 1) and importantly, were obtained with minimal evaporative loss during sampling. These data are generally consistent with MMADs reported in other studies that measured PSD using a LFCI, e.g., Alderman et al. (0.5–0.6 μm), Oldham et al. (0.9–1.2 μm), Kane and Li (0.5–0.9 μm) for various e-cigarette devices, and Pourchez et al. (0.7–1.2 μm) for various e-liquids that were aerosolized at different power settings (4, 35, 36, 58). In contrast, Mikheev et al. reported MMADs of ~0.3 μm for several different flavored e-liquids and Baassiri et al. reported MMADs that ranged from 2.3 to 3.6 μm (7, 8). It is important to note that it is difficult to inter-compare measurement results among studies because other factors that could influence PSD such as e-liquid composition (including purities and impurities in commercial products) and e-cigarette generation and device power settings were not standardized among studies in the literature.

Despite significant differences in PSD determined among e-liquid formulations (Table 1), the modeled regional and total aerosol particle deposition fractions in the respiratory tract were similar (Figure 4). Baassari et al. also noted that despite influences of certain factors on aerosol PSD, these variations might not translate to clinically important differences in lung deposition patterns (7). Hence, when modeling particle lung deposition, future efforts should be placed on improving understanding of those consumable-, device-, and user-related factors that have large impacts on PSD (i.e., sufficient to alter estimates of respiratory tract deposition).

MMADs calculated from the SMPS data (Table 2) tended to be smaller for e-liquids prepared using 70:30 PG:VG compared with e-liquids prepared using 30:70 PG:VG. PG has a lower boiling point and higher vapor pressure compared with VG, so more of the 70:30 PG:VG e-liquids evaporated, yielding relatively smaller MMADs. For the e-liquids with prepared with 30:70 PG:VG, the higher boiling point and vapor pressure of VG would translate into less evaporation, yielding relatively larger MMAD values. In general, MMADs calculated from the SMPS data (Table 2) tended to be similar or larger to MMADs measured without dilution using a LFCI (Table 1). This observation was somewhat surprising given the need for dilution air to sample aerosol using the SMPS and likely reflects the larger GSD of the SMPS data (~2.5 for all e-liquids) compared with the GSD of the LFCI data (~1.4 for all e-liquids). From the Hatch-Choate equation (Equation 1), conversion of CMD values from the SMPS to MMD values depends on Ln(GSD)^2^, so a larger GSD will yield a larger calculated MMD value. All MMADs calculated from the FMPS data were below 75 nm and were monodisperse, i.e., GSDs < 1.005 (Table 2). Both the FMPS and SMPS sampled diluted aerosol with sheath flow of, respectively, 40 and 10 LPM, though the inlet sampling flowrate of the FMPS was 10 LPM compared with the SMPS at 0.3 LPM, which resulted in more dilution (400× compared with 3× ) of the sampled aerosol (Figure 1). These data illustrate that the high dilution of the e-cigarette aerosol necessary for measurement using an FMPS and the high sampling flow rate of this instrument resulted in extensive evaporation independent of humectant composition to the point where only the non-volatile components of droplets remained during measurement. Previously, Ingebrethsen et al. reported that average particle diameters determined for e-cigarette aerosols by an electrical mobility sizer were ~50 nm, which was believed to result from almost complete particle evaporation at the dilution levels and conditions of their measurements (60). Mikheev et al. (63) also observed that e-cigarette aerosol particles contained both volatile and less volatile compounds and when the volatile compounds evaporated at high sampling flow rates and dilution using a mobility sizer, the remaining particles were nanoparticles.

Based on our dosimetry modeling using the LFCI data, it was estimated that ~75% of aerosol particles breathed in by a user could be exhaled and serve as a source of secondhand exposure (Figure 4). Similarly, Sundahl et al. (68) modeled nicotine deposition in the respiratory tract of e-cigarette users and reported that 75–90% of nicotine droplets would be exhaled into the surrounding atmosphere. Dosimetry studies on secondhand exposure to e-cigarette aerosols are scarce (6). One study used a smoking machine to generate aerosol from an e-cigarette and measured PSD *in situ* using laser diffraction. The authors used the PSD data to model passive exposure and predicted total respiratory deposition of 15–30% (of which, 7–10% was in the P region) to a bystander (69). Note that in this study design, changes in aerosol PSD during inhalation and exhalation were not captured by using a smoking machine. Another study had e-cigarette users exhale aerosol in a room and measured PSD at a distance of 2 meters away using a FMPS to simulate a bystander; based on size distribution measurements, the authors predicted 20% of particles would deposit in the H region, 32% in the TB region, and 48% in the P region of a passively exposed person (70). As demonstrated in the current study, e-cigarette aerosol size values determined using a FMPS were likely smaller than *in situ* because of extensive evaporation of volatile constituents during sampling, which in turn, could yield higher estimates of regional particle deposition throughout the respiratory tract. Regardless, the data presented herein, and these cited studies conceptually support the potential for secondhand exposure from e-cigarettes to bystanders in homes and employees in workplace settings; however, estimates of magnitude should be interpreted with caution because aerosol PSD input to these dosimetry models were strongly influenced by the choice of sampling method and modeling required the assumption that e-cigarette aerosol behaved as non-volatile particles.

### Study Limitations

The current study used MPPD to conceptually demonstrate particle deposition in the respiratory tract of an e-cigarette user and estimate the exhaled fraction, which could serve as a source of secondhand exposure to bystanders. It is important to understand that models for non-volatile aerosols such as MPPD are not suitable for making accurate predictions of e-cigarette deposition in the respiratory tract (69). Recently, investigators have developed dynamic models for e-cigarette aerosols that contain volatile constituents (19, 20, 59). These dynamic models account for the high number concentration produced by an e-cigarette puff, the cloud behavior of aerosol that can change PSD *via* coagulation during mouth hold (i.e., increase in particle size and decrease in particle number), hygroscopic growth of droplets from absorption of water in the humid environment of the lung, conductive heat and diffusive/convective vapor transport of volatile constituents, and dilution/mixing in residual air in the lung as particles travel throughout the respiratory tract. Additionally, droplet chemistry is an important factor because the thermodynamics of vapor-liquid partitioning of constituents has a major influence on the deposition characteristics of vapor constituents in the respiratory tract. Several studies have demonstrated that droplet chemistry varies with PG:VG ratio (54, 71, 72). Hence, for e-cigarette aerosol, dosimetry modeling based on PSD alone is insufficient to accurately predict the fate of inhaled particles (59).

By accounting for both physical and chemical factors, dynamic models can be applied to realistic scenarios for e-cigarette use, i.e., puff withdrawal into the oral cavity, mouth hold, dilution of the e-cigarette puff in the mouth with the subsequent dilution from inhaled air, inhalation of the diluted puff into the lower respiratory tract, lung-hold, and exhalation of aerosol into the surrounding air (19, 59). Results of dynamic modeling indicated that particle size gets progressively larger as aerosol travels from puff withdrawal from the e-cigarette into the oral cavity (smallest particle size) to after puff to mouth hold to reaching the alveolar region to exhaled fraction (largest particle size); the smallest particles will coagulate during the puffing and mouth hold phases and grow in size (19, 20, 59). As these particles travel through the lungs, their size will continue to grow from absorption of water in the humid lung, and the net effect is that exhaled particles will be larger than inhaled particles (19, 20, 59). This growth in particle size has implications for dosimetry modeling for e-cigarette users and for secondhand exposure potential. For example, modeling by Asgharian et al. indicated that for a 1 μm e-cigarette aerosol particle (similar to the size reported in Table 1), total deposition calculated using a dynamic model was ~85%; however, the total deposition predicted using an insoluble particle model was ~35%. For the P region, the dynamic model predicted 65% particle deposition, whereas the insoluble model predicted 25% particle deposition (19). Assuming that all the non-deposited particles are exhaled, the dynamic model indicated ~15% of particles would be available for secondhand exposure, whereas the insoluble model indicated 65% of particles could be a source of secondhand exposure (similar to our results presented in Figure 4). Pichelstorfer et al. (20) also compared dynamic and insoluble particle models and reported that dynamic models yielded median number-based and mass-based particle sizes that were 242 and 466% larger than situations where dynamics were ignored, which again means that the actual amount deposited would be higher than predicted using a non-volatile particle model. The primary reason for this difference in deposition predictions between dynamic and insoluble models is the volatility of e-cigarette aerosol constituents and the corresponding contribution to deposition from vapor uptake, which were not accounted for in the insoluble particle models (19). Currently, these dynamic models are not yet widely available for use by the research community though it is expected that in the future, if available, their use will be adopted to improve dosimetry estimates.

Finally, it is worth noting that in the current work, we only focused on the influence of consumable-related factors on aerosol PSD, though it is recognized that device- and user-related factors can also influence aerosol particle size. For example, PSD is reported to be influenced by device coil power setting and temperature (4, 33, 39, 62) as well as puff flow rate (8, 35, 73).

## Summary

Laboratory-prepared e-liquids and a second-generation reference e-cigarette were used to test the hypothesis that e-liquid composition (humectant ratio, flavorings, and nicotine) influenced aerosol PSD. Results from LFCI measurements demonstrated that the proportion of humectants and the presence of nicotine or flavorings significantly influenced MMAD values; however, these differences did not translate into meaningful differences in estimates of regional particle deposition throughout the respiratory tract of e-cigarette users. Notably, use of a LFCI permitted determination of mass-based MMADs with minimal bias from evaporation during sampling. In contrast, monitoring using a FMPS demonstrated significant evaporation of volatile aerosol particle constituents, which yielded PSDs that were an order of magnitude smaller than the native state of droplets produced during puffing. This improved method to characterize physical properties of volatile aerosol particles yielded MMAD values more representative of e-cigarette aerosol *in situ*, which in turn, can help to improve validity of size distribution values input to dosimetry models to estimate exposures to users and bystanders. Particle deposition modeling assuming non-volatile particles conceptually supported the premise that e-cigarettes can be a source of secondhand exposure to persons in proximity to users, whether at home, in a vehicle, or in a workplace. Use of a model developed for non-volatile aerosol particles precluded accurate estimation of the magnitude of aerosol deposition in the respiratory tract of users and the exhaled fraction that could serve a source of secondhand exposure. As dynamic models become more readily available to researchers, understanding of the fate of aerosol generated by e-cigarettes will improve.

## Data Availability Statement

The original contributions presented in the study are included in the article/Supplementary Material, further inquiries can be directed to the corresponding author.

## Author Contributions

AR, AS, and RL contributed to conception and design of the study. AR performed the statistical analysis under guidance from MV. AR and AS wrote the first draft of the manuscript. All authors contributed to manuscript revision, read, and approved the submitted version.

## Author Disclaimer

The findings and conclusions in this report are those of the authors and do not necessarily represent the official position of the National Institute for Occupational Safety and Health, Centers for Disease Control and Prevention.

## Conflict of Interest

The authors declare that the research was conducted in the absence of any commercial or financial relationships that could be construed as a potential conflict of interest.

## Publisher's Note

All claims expressed in this article are solely those of the authors and do not necessarily represent those of their affiliated organizations, or those of the publisher, the editors and the reviewers. Any product that may be evaluated in this article, or claim that may be made by its manufacturer, is not guaranteed or endorsed by the publisher.

## References

[1] Bhatnagar A Whitsel LP Ribisl KM Bullen C Chaloupka F Piano MR. Electronic cigarettes: a policy statement from the American Heart Association. Circulation. (2014) 130:1418–36. 10.1161/CIR.0000000000000107 25156991 PMC7643636

[2] Schmidt S. Vaper, beware: the unique toxicological profile of electronic cigarettes. Environ Health Perspect. (2020) 128:052001. 10.1289/EHP6628 32363917 PMC7263459

[3] Schraufnagel DE Blasi F Drummond MB Lam DC Latif E Rosen MJ. Electronic cigarettes. A position statement of the forum of international respiratory societies. Am J Respir Crit Care Med. (2014) 190:611–8. 10.1164/rccm.201407-1198PP 25006874

[4] Pourchez J Parisse S Sarry G Perinel-Ragey S Vergnon JM Clotagatide A. Impact of power level and refill liquid composition on the aerosol output and particle size distribution generated by a new-generation e-cigarette device. Aerosol Sci Technol. (2018) 52:359–69. 10.1080/02786826.2017.1422857

[5] Protano C Avino P Manigrasso M Vivaldi V Perna F Valeriani F. Environmental electronic vape exposure from four different generations of electronic cigarettes: airborne particulate matter levels. Int J Environ Res Public Health. (2018) 15:2172. 10.3390/ijerph15102172 30282910 PMC6210766

[6] Stefaniak AB LeBouf RF Ranpara AC Leonard SS. Toxicology of flavoring- and cannabis-containing e-liquids used in electronic delivery systems. Pharmacol Ther. (2021) 224:107838. 10.1016/j.pharmthera.2021.107838 33746051 PMC8251682

[7] Baassiri M Talih S Salman R Karaoghlanian N Saleh R El Hage R. Clouds and “throat hit”: Effects of liquid composition on nicotine emissions and physical characteristics of electronic cigarette aerosols. Aerosol Sci Technol. (2017) 51:1231–9. 10.1080/02786826.2017.1341040 32863527 PMC7453347

[8] Mikheev VB Ivanov A Lucas EA South PL Colijn HO Clark PI. Aerosol size distribution measurement of electronic cigarette emissions using combined differential mobility and inertial impaction methods: smoking machine and puff topography influence. Aerosol Sci Technol. (2018) 52:1233–48. 10.1080/02786826.2018.1513636 32773918 PMC7409981

[9] El-Hellani A Salman R El-Hage R Talih S Malek N Baalbaki R. Nicotine and carbonyl emissions from popular electronic cigarette products: correlation to liquid composition and design characteristics. Nicotine Tob Res. (2018) 20:215–23. 10.1093/ntr/ntw280 27798087 PMC5896517

[10] Bonner E Chang Y Christie E Colvin V Cunningham B Elson D. The chemistry and toxicology of vaping. Pharmacol Therap. (2021) 225:e107837. 10.1016/j.pharmthera.2021.107837 33753133 PMC8263470

[11] Soulet S Duquesne M Toutain J Pairaud C Lalo H. Experimental method of emission generation calibration based on reference liquids characterization. Int J Environ Res Public Health. (2019) 16:2262. 10.3390/ijerph16132262 31248048 PMC6651204

[12] Girvalaki C Tzatzarakis M Kyriakos CN Vardavas AI Stivaktakis PD Kavvalakis M. Composition and chemical health hazards of the most common electronic cigarette liquids in nine European countries. Inhal Toxicol. (2018) 30:361–9. 10.1080/08958378.2018.1527879 30369275

[13] Havermans A Krusemann EJZ Pennings J de Graaf K Boesveldt S Talhout R. Nearly 20 000 e-liquids and 250 unique flavour descriptions: an overview of the Dutch market based on information from manufacturers. Tob Control. (2021) 30:57–62. 10.1136/tobaccocontrol-2019-055303 31685584 PMC7803909

[14] Krüsemann EJZ Boesveldt S de Graaf K Talhout R. An E-liquid flavor wheel: a shared vocabulary based on systematically reviewing E-liquid flavor classifications in literature. Nicotine Tob Res. (2019) 21:1310–9. 10.1093/ntr/nty101 29788484 PMC6751518

[15] El-Hellani A El-Hage R Baalbaki R Salman R Talih S Shihadeh A. Free-base and protonated nicotine in electronic cigarette liquids and aerosols. Chem Res Toxicol. (2015) 28:1532–7. 10.1021/acs.chemrestox.5b00107 26158618 PMC4920054

[16] Harvanko AM Havel CM Jacob P Benowitz NL. Characterization of nicotine salts in 23 electronic cigarette refill liquids. Nicotine Tob Res. (2020) 22:1239–43. 10.1093/ntr/ntz232 31821492 PMC7291795

[17] Pankow JF Duell AK Peyton DH. Free-base nicotine fraction α(fb) in non-aqueous versus aqueous solutions: electronic cigarette fluids without versus with dilution with water. Chem Res Toxicol. (2020) 33:1729–35. 10.1021/acs.chemrestox.0c00008 32255343 PMC9968495

[18] Romberg AR Miller Lo EJ Cuccia AF Willett JG Xiao H Hair EC. Patterns of nicotine concentrations in electronic cigarettes sold in the United States, 2013-2018. Drug Alcohol Depend. (2019) 203:1–7. 10.1016/j.drugalcdep.2019.05.029 31386973 PMC6765364

[19] Asgharian B Rostami AA Price OT Pithawalla YB. Regional deposition of inhaled aerosol constituents from Electronic Nicotine Delivery Systems (ENDS) in the respiratory tract. J Aerosol Sci. (2018) 126:7–20. 10.1016/j.jaerosci.2018.08.006

[20] Pichelstorfer L Winkler-Heil R Boy M Hofmann W. Aerosol dynamics simulations of the anatomical variability of e-cigarette particle and vapor deposition in a stochastic lung. J Aerosol Sci. (2021) 158:105706. 10.1016/j.jaerosci.2020.105706

[21] Bhatt JM Ramphul M Bush A. An update on controversies in e-cigarettes. Paediatr Respir Rev. (2020) 36:75–86. 10.1016/j.prrv.2020.09.003 33071065 PMC7518964

[22] Protano C Manigrasso M Avino P Vitali M. Second-hand smoke generated by combustion and electronic smoking devices used in real scenarios: ultrafine particle pollution and age-related dose assessment. Environ Int. (2017) 107:190–5. 10.1016/j.envint.2017.07.014 28750224

[23] Tzortzi A Teloniatis S Matiampa G Bakelas G Tzavara C Vyzikidou VK. Passive exposure of non-smokers to E-Cigarette aerosols: sensory irritation, timing and association with volatile organic compounds. Environ Res. (2020) 182:108963. 10.1016/j.envres.2019.108963 31837549

[24] Chen R Aherrera A Isichei C Olmedo P Jarmul S Cohen JE. Assessment of indoor air quality at an electronic cigarette (Vaping) convention. J Expo Sci Environ Epidemiol. (2018) 28:522–9. 10.1038/s41370-017-0005-x 29288255

[25] Johnson JM Naeher LP Yu X Sosnoff C Wang L Rathbun SL. A biomonitoring assessment of secondhand exposures to electronic cigarette emissions. Int J Hyg Environ Health. (2019) 222:816–23. 10.1016/j.ijheh.2019.04.013 31085112 PMC6938228

[26] Li L Nguyen C Lin Y Guo Y Fadel NA Zhu Y. Impacts of electronic cigarettes usage on air quality of vape shops and their nearby areas. Sci Total Environ. (2021) 760:143423. 10.1016/j.scitotenv.2020.143423 33162144 PMC7937385

[27] Logue JM Sleiman M Montesinos VN Russell ML Litter MI Benowitz NL. Emissions from electronic cigarettes: assessing vapers' intake of toxic compounds, secondhand exposures, and the associated health impacts. Environ Sci Technol. (2017) 51:9271–9. 10.1021/acs.est.7b00710 28766331

[28] Soule EK Maloney SF Spindle TR Rudy AK Hiler MM Cobb CO. Electronic cigarette use and indoor air quality in a natural setting. Tob Control. (2017) 26:109–12. 10.1136/tobaccocontrol-2015-052772 26880745 PMC4985441

[29] Tigova O Amalia B Castellano Y Fu M Nogueira SO Kyriakos CN. Secondhand exposure to e-cigarette aerosols among smokers: a cross-sectional study in six European countries of the EUREST-PLUS ITC Europe Surveys. Tob Induc Dis. (2018) 16:A11. 10.18332/tid/99117 31516465 PMC6661852

[30] Khachatoorian C Jacob P III Sen A Zhu Y Benowitz NL Talbot P. Identification and quantification of electronic cigarette exhaled aerosol residue chemicals in field sites. Environ Res. (2019) 170:351–8. 10.1016/j.envres.2018.12.027 30623881 PMC6410739

[31] Melstrom P Koszowski B Thanner MH Hoh E King B Bunnell R. Measuring PM2.5. ultrafine particles, nicotine air and wipe samples following the use of electronic cigarettes. Nicotine Tobacco Res. (2017) 19:1055–61. 10.1093/ntr/ntx058 28340080

[32] Kleinstreuer C Feng Y. Lung deposition analyses of inhaled toxic aerosols in conventional and less harmful cigarette smoke: a review. Int J Environ Res Public Health. (2013) 10:4454–85. 10.3390/ijerph10094454 24065038 PMC3799535

[33] Lechasseur A Altmejd S Turgeon N Buonanno G Morawska L Brunet D. Variations in coil temperature/power and e-liquid constituents change size and lung deposition of particles emitted by an electronic cigarette. Physiol Rep. (2019) 7:e14093. 10.14814/phy2.14093 31140749 PMC6540444

[34] Li Y Cui H Chen L Fan M Cai J Guo J. Modeled respiratory tract deposition of smoke aerosol from conventional cigarettes, electronic cigarettes and heat-not-burn products. Aerosol Air Qual Res. (2021) 21:241. 10.4209/aaqr.200241

[35] Oldham MJ Zhang J Rusyniak MJ Kane DB Gardner WP. Particle size distribution of selected electronic nicotine delivery system products. Food Chem Toxicol. (2018) 113:236–40. 10.1016/j.fct.2018.01.045 29408542

[36] Alderman SL Song C Moldoveanu SC Cole SK. Particle size distribution of e-cigarette aerosols and the relationship to cambridge filter pad collection efficiency. Beitrage zur Tabakforschung Int/Contrib Tobacco Res. (2014) 26:183–90. 10.1515/cttr-2015-0006

[37] Zervas E Litsiou E Konstantopoulos K Poulopoulos S Katsaounou P. Physical characterization of the aerosol of an electronic cigarette: impact of refill liquids. Inhal Toxicol. (2018) 30:218–23. 10.1080/08958378.2018.1500662 30257112

[38] Zhang Y Sumner W Chen DR. *In vitro* particle size distributions in electronic and conventional cigarette aerosols suggest comparable deposition patterns. Nicotine Tob Res. (2013) 15:501–8. 10.1093/ntr/nts165 23042984

[39] Mulder HA Patterson JL Halquist MS Kosmider L Turner JBMG Poklis JL. The effect of electronic cigarette user modifications and E-liquid adulteration on the particle size profile of an aerosolized product. Sci Rep. (2019) 9:10221. 10.1038/s41598-019-46387-2 31308389 PMC6629610

[40] Prévôt N de Oliveira F Perinel-Ragey S Basset T Vergnon JM Pourchez J. Nicotine delivery from the refill liquid to the aerosol *via* high-power e-cigarette device. Sci Rep. (2017) 7:2592. 10.1038/s41598-017-03008-0 28572636 PMC5453927

[41] Larcombe AN Janka MA Mullins BJ Berry LJ Bredin A Franklin PJ. The effects of electronic cigarette aerosol exposure on inflammation and lung function in mice. Am J Physiol Lung Cell Mol Physiol. (2017) 313:L67–79. 10.1152/ajplung.00203.2016 28360111

[42] Manigrasso M Buonanno G Fuoco FC Stabile L Avino P. Electronic cigarettes: age-specific generation-resolved pulmonary doses. Environ Sci Pollut Res. (2017) 24:13068–79. 10.1007/s11356-017-8914-8 28382447

[43] Belka M Lizal F Jedelsky J Jicha M Pospisil J. Measurement of an electronic cigarette aerosol size distribution during a puff. In: Paper Presented at the EPJ Web of Conferences. Marienbad (2017).

[44] Bertholon JF Becquemin MH Roy M Roy F Ledur D Annesi Maesano I. Comparison of the aerosol produced by electronic cigarettes with conventional cigarettes and the shisha. Rev Mal Respir. (2013) 30:752–7. 10.1016/j.rmr.2013.03.003 24267765

[45] Fuoco FC Buonanno G Stabile L Vigo P. Influential parameters on particle concentration and size distribution in the mainstream of e-cigarettes. Environ Pollut. (2014) 184:523–9. 10.1016/j.envpol.2013.10.010 24172659

[46] Laube BL Afshar-Mohajer N Koehler K Chen G Lazarus P Collaco JM. Acute and chronic *in vivo* effects of exposure to nicotine and propylene glycol from an E-cigarette on mucociliary clearance in a murine model. Inhal Toxicol. (2017) 29:197–205. 10.1080/08958378.2017.1336585 28651446 PMC5553614

[47] Manigrasso M Buonanno G Fuoco FC Stabile L Avino P. Aerosol deposition doses in the human respiratory tree of electronic cigarette smokers. Environ Pollut. (2015) 196:257–67. 10.1016/j.envpol.2014.10.013 25463721

[48] Lee MS LeBouf RF Son YS Koutrakis P Christiani DC. Nicotine, aerosol particles, carbonyls and volatile organic compounds in tobacco- and menthol-flavored e-cigarettes. Environ Health. (2017) 16:42. 10.1186/s12940-017-0249-x 28449666 PMC5406907

[49] Cheah NP Chong NW Tan J Morsed FA Yee SK. Electronic nicotine delivery systems: regulatory and safety challenges: Singapore perspective. Tob Control. (2014) 23:119–25. 10.1136/tobaccocontrol-2012-050483 23204074

[50] Szafran BN Pinkston R Perveen Z Ross MK Morgan T Paulsen DB. Electronic-cigarette vehicles and flavoring affect lung function and immune responses in a murine model. Int J Mol Sci. (2020) 21:6022. 10.3390/ijms21176022 32825651 PMC7504509

[51] Jackler RK Ramamurthi D. Nicotine arms race: JUUL and the high-nicotine product market. Tob Control. (2019) 28:623–8. 10.1136/tobaccocontrol-2018-054796 30733312

[52] Tierney PA Karpinski CD Brown JE Luo W Pankow JF. Flavour chemicals in electronic cigarette fluids. Tob Control. (2016) 25:e10–15. 10.1136/tobaccocontrol-2014-052175 25877377 PMC4853541

[53] Kim JJ Sabatelli N Tutak W Giuseppetti A Frukhtbeyn S Shaffer I. Universal electronic-cigarette test: physiochemical characterization of reference e-liquid. Tob Induc Dis. (2017) 15:14. 10.1186/s12971-017-0119-x 28239329 PMC5314484

[54] Ooi BG Dutta D Kazipeta K Chong NS. Influence of the E-cigarette emission profile by the ratio of glycerol to propylene glycol in E-liquid composition. ACS Omega. (2019) 4:13338–48. 10.1021/acsomega.9b01504 31460462 PMC6705204

[55] (AFNOR) AFdN. Cigarettes Electroniques et E-Liquides. Partie 3: Exigences et Méthodes D'essais Relatives aux Emissions. Paris: AFNOR (2016). p. 46.

[56] NIDA. NIDA Standard Drug Program. (2021). Available online at: https://www.drugabuse.gov/research/research-data-measures-resources/nida-drug-supply-program/ (accessed July 15, 2021).

[57] CORESTA. Recommended Method No. 81. CORESTA, Paris, France (2015).

[58] Kane DB Li W. Particle size measurement of electronic cigarette aerosol with a cascade impactor. Aerosol Sci Technol. (2021) 55:205–14. 10.1080/02786826.2020.1849536

[59] Asgharian B Price OT Rostami AA Pithawalla YB. Deposition of inhaled electronic cigarette aerosol in the human oral cavity. J Aerosol Sci. (2018) 116:34–47. 10.1016/j.jaerosci.2017.11.01434392198

[60] Ingebrethsen BJ Cole SK Alderman SL. Electronic cigarette aerosol particle size distribution measurements. Inhal Toxicol. (2012) 24:976–84. 10.3109/08958378.2012.744781 23216158

[61] Biswas P Jones CL Flagan RC. Distortion of size distributions by condensation and evaporation in aerosol instruments. Aerosol Sci Technol. (1987) 7:231–46. 10.1080/02786828708959161

[62] Floyd EL Queimado L Wang J Regens JL Johnson DL. Electronic cigarette power affects count concentration and particle size distribution of vaping aerosol. PLoS ONE. (2018) 13:e0210147. 10.1371/journal.pone.0210147 30596800 PMC6312322

[63] Mikheev VB Brinkman MC Granville CA Gordon SM Clark PI. Real-time measurement of electronic cigarette aerosol size distribution and metals content analysis. Nicotine Tob Res. (2016) 18:1895–902. 10.1093/ntr/ntw128 27146638 PMC4978987

[64] Sosnowski TR Odziomek M. Particle size dynamics: toward a better understanding of electronic cigarette aerosol interactions with the respiratory system. Front Physiol. (2018) 9:853. 10.3389/fphys.2018.00853 30038580 PMC6046408

[65] O'Shaughnessy PT Raabe OG. A comparison of cascade impactor data reduction methods. Aerosol Sci Technol. (2003) 37:187–200. 10.1080/02786820300956

[66] Ebersole J Samburova V Son Y Cappelli D Demopoulos C Capurro A. Harmful chemicals emitted from electronic cigarettes and potential deleterious effects in the oral cavity. Tob Induc Dis. (2020) 18:41. 10.18332/tid/116988 32435175 PMC7233525

[67] Rouabhia M Piché M Corriveau MN Chakir J. Effect of e-cigarettes on nasal epithelial cell growth, Ki67 expression, and pro-inflammatory cytokine secretion. Am J Otolaryngol. (2020) 41:102686. 10.1016/j.amjoto.2020.102686 32866847

[68] Sundahl M Berg E Svensson M. Aerodynamic particle size distribution and dynamic properties in aerosols from electronic cigarettes. J Aerosol Sci. (2017) 103:141–50. 10.1016/j.jaerosci.2016.10.009

[69] Sosnowski TR Kramek-Romanowska K. Predicted deposition of E-cigarette aerosol in the human lungs. J Aerosol Med Pulm Drug Deliv. (2016) 29:299–309. 10.1089/jamp.2015.1268 26907696

[70] Protano C Manigrasso M Avino P Sernia S Vitali M. Second-hand smoke exposure generated by new electronic devices (IQOS^®^ and e-cigs) and traditional cigarettes: submicron particle behaviour in human respiratory system. Ann Ig. (2016) 28:109–12. 10.7416/ai.2016.2089 27071321

[71] Conklin DJ Ogunwale MA Chen Y Theis WS Nantz MH Fu XA. Electronic cigarette-generated aldehydes: the contribution of e-liquid components to their formation and the use of urinary aldehyde metabolites as biomarkers of exposure. Aerosol Sci Technol. (2018) 52:1219–32. 10.1080/02786826.2018.1500013 31456604 PMC6711607

[72] Jensen RP Strongin RM Peyton DH. Solvent chemistry in the electronic cigarette reaction vessel. Sci Rep. (2017) 7:2549. 10.1038/srep42549 28195231 PMC5307352

[73] Fisenko SP Rostami AA Kane DB Pithawalla YB Maximoff SN Li W. Model of aerosol evolution in high supersaturated glycerol-air vapor mixtures. Aerosol Sci Technol. (2021) 55:871–85. 10.1080/02786826.2021.1904130

