# Supplementary material for: Influence of E-Liquid Humectants, Nicotine, and Flavorings on Aerosol Particle Size Distribution and Implications for Modeling Respiratory Deposition
Source: Front Public Health. 2022 Mar 17;10:782068. doi: 10.3389/fpubh.2022.782068 (PMC8968757; doi:10.3389/fpubh.2022.782068)
Supplement: Supplementary file 1 [file Data_Sheet_1.docx]

**Supplemental Material**

# **Influence of e-liquid humectants, nicotine, and flavorings on aerosol particle size distribution and implications for modeling respiratory deposition**

**Aleksandr B. Stefaniak, Anand C. Ranpara, M. Abbas Virji, Ryan F. LeBouf***

Respiratory Health Division, National Institute for Occupational Safety and Health, Morgantown, WV

***corresponding author**

**Dr. Ryan F. LeBouf**

Respiratory Health Division

National Institute for Occupational Safety and Health

Morgantown, WV, 26505

Tel.: +1-304-285-6287

E-mail: [igu6@cdc.gov](mailto:igu6@cdc.gov)

Disclaimer: The findings and conclusions in this report are those of the authors and do not necessarily represent the official position of the National Institute for Occupational Safety and Health, Centers for Disease Control and Prevention.


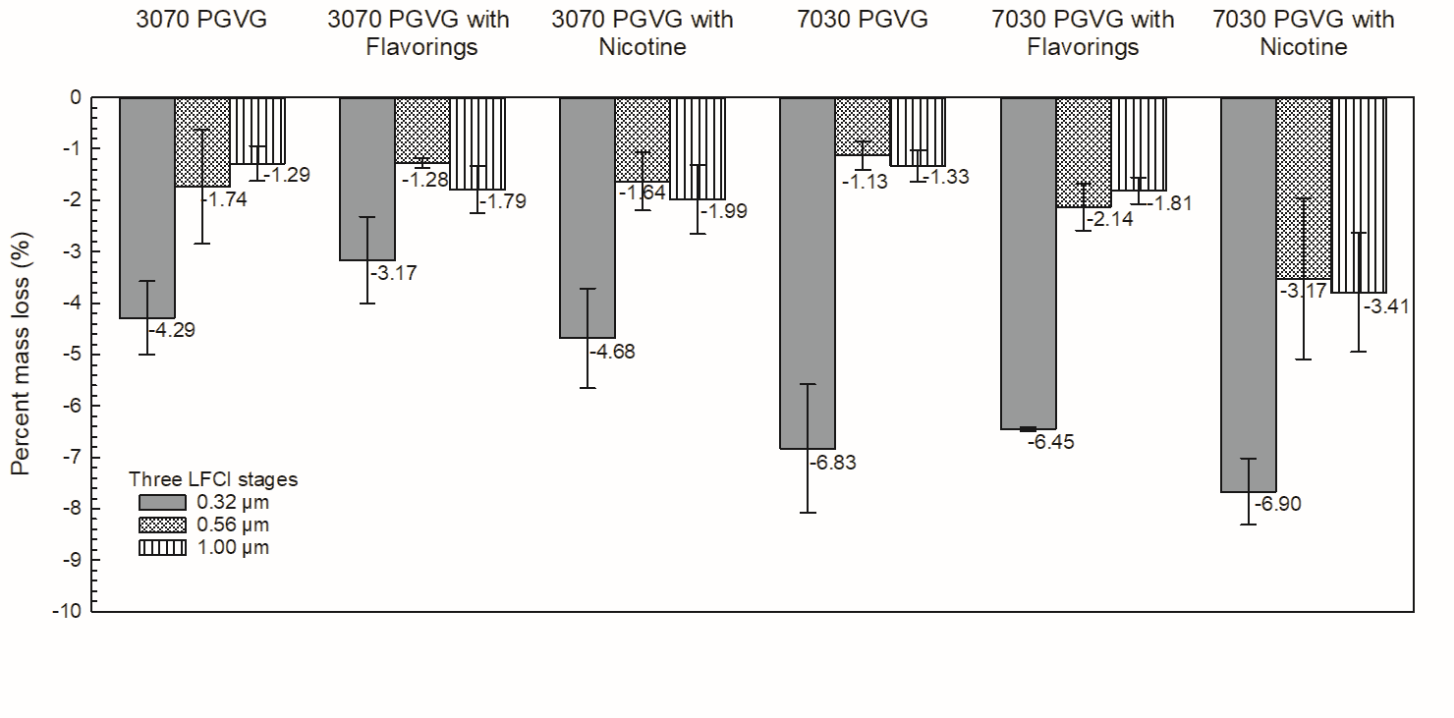


Figure S1. Percent mass loss by e-liquid for three low flow cascade impactor stages demonstrating minimal evaporation during sampling and gravimetric analysis. PG = propylene glycol, VG = vegetable glycerin.

Table S1. Physiological parameters for Yeh/Schum symmetrical lung model (MPPD)

| MPPD Parameters | Values |
| --- | --- |
| Functional residual capacity (ml) | 3300 |
| Head volume (ml) | 50 |
| Breathing interval | Single breath |
| Breathing Frequency per minute | 12 |
| Tidal Volume (ml) | 625 |
| Inspiratory Fraction | 0.5 |
| Breathing scenario | Oronasal-mouth |
